# Current Understanding and Future Challenges in Physical Activity during Pregnancy

**DOI:** 10.3390/jcm12123986

**Published:** 2023-06-12

**Authors:** Lulu Wang, Yanting Wu

**Affiliations:** Obstetrics and Gynecology Hospital, Institute of Reproduction and Development, Fudan University, Shanghai 200011, China; wanglulu10040@fckyy.org.cn

Obesity and overweight attributed to poor nutrition and a lack of physical activity increasingly become a serious problem among women of reproductive age [1]. Pregnancy is considered to be a valuable window of opportunity for maternal and offspring well-being improvements by lifestyle modification, based on the developmental origins of health and disease (DOHaD) theory [2]. However, physical inactivity as well as an unbalanced diet are rife during pregnancy.

As recommended by the World Health Organization (WHO) and the Royal College of Obstetricians and Gynecologists (RCOG), sedentary time should be reduced and a weekly minimum of 150 min of aerobic physical activity of moderate intensity is needed for pregnant women [3]. In a systematic review of 44 studies, it was found that physical activity levels were below the international recommendations in nearly 60% of articles [4]. Possible reasons are physically inactiveness caused by pregnancy-related symptoms, busy schedules and lack of knowledge about safety and benefits of exercise during pregnancy [3].

Previous studies have explored the impact and safety of different varieties of sports in pregnant women, either using a virtual program or via on-site supervision [5,6]. Generally, sports can be classified into the following four categories: endurance (aerobic, running, biking, etc.), athletic (climbing, karate, etc.), light (aqua gymnastics, Pilates, yoga, etc.) and combined (badminton, dancing, etc.) [7]. Aerobic, resistance and combined sports were the most studied among pregnant women [8]. In addition, therapeutic aquatic exercise, yoga and Pilates are gaining more and more attention for research [9,10]. The exercise programs designed in most studies were characterized with moderate intensity, commencement in the second trimester, a duration of 20–60 min and a frequency of 1–3 sessions per week [10]. As for study populations, obese/overweight women as well as healthy women were mostly targeted, while those with high-risk pregnancies such as assisted reproduction technologies (ART) and an advanced age were less studied [3,10]. The instrument used for the recording and measurement of physical activity plays a pivotal role in outcome analysis. The most widely applied tools are the International Physical Activity Questionnaire (IPAQ), Pregnancy Physical Activity Questionnaire (PPAQ) [11], Global Physical Activity Questionnaire (GPAQ) [9] for self-reported exercise data, Borg scale for perception of effort, and metabolic equivalent of task (MET) for intensity measurements and recording of the amount of exercise.

The short- and long-term influence of pregnancy physical activity on maternal and offspring health have always been a heated topic in this area. Previous systematic reviews and meta-analyses supported a positive effect of physical activity for preventing the onset of gestational diabetes mellitus (GDM), hypertensive disorders of pregnancy (HDP) and excessive gestational weight gain [6,8,9]. Moreover, a beneficial impact of yoga and aquatic exercise on preventing perinatal depression and anxiety was also noticed [9,10]. In terms of obstetric outcomes, prenatal physical activity has the potential to reduce the rates of cesarean section and preterm delivery [12]. A randomized clinical trial conducted in 2020 also found that a supervised online exercise program starting in the early pregnancy stages significantly reduced the prevalence of perineal tears and episiotomy rates, showing potential benefits for preventing pelvic floor dysfunction and urinary incontinence postpartum in the long run [12,13]. In terms of the impact on fetal–infant health, it is suggested that gestational exercise is likely to reduce the risk of small-for-gestational age and macrosomia at birth, less admissions to neonatal intensive care units and childhood overweight/obesity during the first year of age [5,14]. Many studies have assessed the association between pregnancy exercise and birthweight, but current evidence remains to be inconclusive. Interestingly, a systematic review that evaluated levels of physical activity in different pregnant populations worldwide indicated that the current levels women were achieving were too low to solidly prove maternal and offspring benefits in clinical research [4].

The safety of physical activity during pregnancy possibly worries mothers most when they have a lack of knowledge regarding the fact that exercise is necessary and beneficial both for them and their child. Thus, it is of great importance to educate women pre-pregnancy or in the early pregnancy stages on the advantages of gestational exercise, especially women who received ART. Women should also be warned about the absolute and relative contraindications to physical activity such as severe cardiac or respiratory diseases in advance. An individualized strategy tailored by professional physicians taking into consideration personal motivation and resistance is recommended for improving adherence to physical activity throughout pregnancy [15]. 

In conclusion, physical activity during pregnancy has great potential to improve maternal and offspring health in different ways. Future clinical studies of higher quality, especially prospective RCTs of larger populations, are warranted for further exploration of more efficient exercises to guide clinical practices.
